# High PEEP Activates ITGB1, Inducing Diaphragm Fibrosis During Prolonged Mechanical Ventilation

**DOI:** 10.3390/biom15101466

**Published:** 2025-10-16

**Authors:** Jiahong Gong, Jianwei Jia, Runze He, Xiaolan Yu, Ye Jiang, Weimin Shen, Xiaoli Qian, Peifeng Xu, Ying Xu, Huiqing Ge

**Affiliations:** Department of Respiratory Care, Regional Medical Center for National Institute of Respiratory Diseases, Sir Run Run Shaw Hospital, School of Medicine, Zhejiang University, Qingchun East Rd. 3, Hangzhou 310016, China; 3322200@zju.edu.cn (J.G.); jiajianwei@zju.edu.cn (J.J.); runzehe@zju.edu.cn (R.H.); 3322199@zju.edu.cn (X.Y.); 3413106@zju.edu.cn (Y.J.); shenweimin@zju.edu.cn (W.S.); qianxiaoli@zju.edu.cn (X.Q.); 3408168@zju.edu.cn (P.X.); 3202014@zju.edu.cn (Y.X.)

**Keywords:** ITGB1, mechanically stimulate, mechanical ventilation, diaphragm fibrosis, TGFβ-1

## Abstract

Background: Mechanical ventilation (MV) with high positive end-expiratory pressure (PEEP) is linked to ventilation-induced diaphragm dysfunction (VIDD), but the role of integrin beta-1 (ITGB1) in PEEP-associated diaphragm fibrosis remains unclear. Methods: Eighteen rabbits were divided into control (CON), MV without PEEP(MV), and MV with 8 cmH_2_O PEEP (PEEP) groups. C2C12 underwent cyclic stretching (15% tension), and ITGB1 was knocked down. Fibrosis markers (TGFβ-1, α-SMA), ITGB1/ROCK1 expression, and pathway activation were analyzed via RNA sequencing, immunohistochemistry, and Western blotting. Results: The PEEP group exhibited elevated airway pressure and upregulated fibrosis markers (TGFβ-1 and α-SMA) alongside activated ITGB1/ROCK1 mechanotransduction pathways. Stretched C2C12 showed morphological shrinkage and increased fibrotic protein expression. RNA sequencing confirmed enrichment in fibrosis- and integrin-related pathways. ITGB1 knockdown attenuated TGFβ-1 and α-SMA induction. Conclusions: ITGB1 mediates PEEP-induced diaphragm fibrosis via TGFβ-1 signaling and collagen deposition, suggesting ITGB1 targeting as a potential therapeutic strategy for VIDD. These findings elucidate the mechanotransduction mechanisms underlying MV-associated diaphragm dysfunction.

## 1. Introduction

The contraction of the diaphragm causes negative pressure in the chest cavity, which is the main source of power during spontaneous respiration. Mechanical ventilation (MV) is a supportive method for maintaining life in critically ill patients with respiratory failure. Positive end-expiratory pressure (PEEP) is an effective component of managing patients undergoing MV. However, about 60–80% of critically ill patients experience diaphragmatic weakness after MV, which is ventilation-induced diaphragmatic dysfunction (VIDD) [1,2].

For critically ill patients, PEEP can improve gas exchange, increase end-expiratory lung volume (EELV), and improve lung homogeneity. However, previous studies have shown that in critically ill patients undergoing high-PEEP mechanical ventilation, the contraction of a single diaphragm fiber can result in a reduced cross-sectional area and shortened fiber length, a condition known as diaphragm fiber atrophy [3,4,5,6].

Considering that high PEEP forces the diaphragm to be compressed and results in geometric changes [4], the diaphragm continues to be subjected to mechanical stress from the lungs [7]. At the cellular level, mechanical stress can affect the mechanical environment of cells and activate intracellular signaling pathways [8], thereby promoting or inhibiting the expression and synthesis of fibrosis-related proteins. During the process of fibrosis, changes in the structure and mechanical properties of tissues can affect the mechanical environment of surrounding cells, thereby affecting their function and behavior. Mechanically sensitive proteins, as important molecules for sensing mechanical forces, play a crucial role in this process.

Our team demonstrated that prolonged application of PEEP (48 h) in mechanically ventilated rabbits will induce collagen deposition and fibrotic changes in the diaphragmatic tissue. Moreover, these structural alterations were associated with activation of the TGF-β1 signaling pathway and promotion of myofibroblast differentiation, suggesting a potential mechanism of this diaphragmatic fibrosis. Research has shown that ITGB1 is associated with TGFβ-1. TGFβ-1 is stored in cells as inactive precursor proteins, including TGFβ-1 and Latency-Associated Peptide (LAP). At this point, due to TGFβ-1 being encapsulated by LAP, preventing it from binding to receptors, TGFβ-1 does not have biological functions. Under mechanical stimulation, ITGB1 can cause dissociation of precursor proteins and promote TGFβ-1 to become an active form, bind to its receptor, and initiate downstream signal transduction pathways [5,9].

ITGB1 is a type of integrin that interacts with the extracellular matrix β subunits, as a bridge between the extracellular matrix and intracellular signaling pathways, playing a role in transmitting and regulating external force signals [10]. The mechanical stress transmission mechanism can affect cell morphology remodeling, cytoskeleton recombination, and gene expression regulation [5]. The effect of mechanical stress can alter the binding affinity between ITGB1 and the extracellular matrix, thereby regulating the force transmission effect between the extracellular matrix and intracellular signaling pathways. In addition, mechanical stress can also affect the expression level and subcellular localization of ITGB1 by altering the activity of intracellular signaling pathways [11,12].

Therefore, we explored whether high PEEP activates mechanosensitive protein ITGB1, enhances collagen accumulation, and stimulates fibrotic remodeling in rabbits with high PEEP by altering the mechanical forces acting on the diaphragm. These findings offer potential novel perspectives on the pathogenesis and therapeutic intervention of VIDD.

## 2. Materials and Methods

### 2.1. Mechanically Ventilated Animal Mode

Fourteen-week-old New Zealand rabbits with a mean body weight of 2.5 kg in the MV and PEEP groups were anesthetized by intraperitoneal injection of 3% sodium pentobarbital (1 mL/kg). Local infiltration of lidocaine was administered at the cervical incision site to provide supplemental analgesia. Moreover, a venous indwelling needle was established under the vein of the rabbit ear. Anesthesia was maintained with an intravenous pentobarbital sodium infusion. A feeding tube was inserted into the stomach via a small incision in the esophagus. The ventilator used in the experiment was the SV800 neonatal circuit ventilator (neonatal type: Mindray, Shenzhen, China). During the experimental period, mechanical ventilation was administered in volume assist/control mode with a tidal volume of 8 mL/kg, with a respiratory rate of 40–50 bpm and the PEEP set at either 0 or 8 cmH_2_O. The end-tidal CO_2_ pressure (PETCO_2_) was monitored at 4 h intervals, and ventilator settings were adjusted accordingly. Ventilator waveforms for pressure and airflow were continuously monitored through the RS-232 output port (RespcareRM, Hangzhou ZhiRuiSi Company, Hangzhou, China), with respiratory parameters recorded in real time. A spontaneous breathing trial (SBT) was performed after 48 h of mechanical ventilation. During this period, parameters such as respiratory rate (RR), tidal volume (VT), and total minute ventilation (VE) were collected. Diaphragmatic ultrasound assessments were performed at baseline, initiation of mechanical ventilation, 48 h timepoint, and during SBT. After discontinuing mechanical ventilation, the diaphragm tissues were collected for subsequent analysis. All animal experimental protocols were conducted in accordance with protocols approved by the Review Committee of Zhejiang University School of Medicine.

### 2.2. Cell Culture and Mechanical Stimulation

C2C12 cells were purchased from Dalian Meilun Biotechnology Co., Ltd. (Dalian, China) and cultured in DMEM (Gibco, NY USA) supplemented with 10% fetal bovine serum (FBS, Cellmax, Beijing, China), 100 U/mL penicillin, and 100 U/mL streptomycin. The cells were incubated at 37°C in a humid environment of 5% CO_2_ and 95% air.

Prior to mechanical stimulation, the elastic cell culture plates were rinsed with PBS and covered with 100 mg/mL poly-lysine solution for 2 h. Next, C2C12 cells were seeded at a 90% confluence. Following adhesion, we started the instruments (celltank, Hangzhou, China) to set a 15% tension rate with cycling loading stimulation for a predetermined duration.

The following gour experimental groups were designed: a control group (CON) without stretching and three groups (1H, 2H, and 4H) subjected to 15% elongation for different time periods (1 h, 2 h, and 4 h) to simulate mechanical overloading.

### 2.3. Knockdown of ITGB1 Expression by siRNA

C2C12 cells were transfected with 50 nM of siRNA targeting ITGB1 (sense: 5′-CCACAGAAGUUUACAUUAA-3′ and antisense: 5′-UUAAUGUAAACUUCUGUGG-3′) using the Lipofectamine RNAiMAX transfection reagent (Invitrogen, Carlsbad, CA, USA). Total RNA and protein were extracted to assess the efficiency of ITGB1 knockdown at both the mRNA and protein levels by qPCR and Western blotting, respectively.

### 2.4. Diaphragm Ultrasonic

Diaphragm activity was assessed using ultrasound measurements. Diaphragmatic motion was evaluated along the costal margin at the midclavicular line, while diaphragmatic thickening was measured at the midaxillary line. End-inspiratory and end-expiratory thickening served as indicators of diaphragmatic function. This approach facilitates a quantitative assessment of diaphragmatic activity during mechanical ventilation.

### 2.5. Ultrastructural Observations

Tissues of the diaphragm were cut into 1–1.5 mm^3^ slices and then fixed in 2.5% glutaraldehyde at 4 °C for 12 h. Then, the samples were fixed, dehydrated, dried, and sectioned according to standard methods. Eventually, the ultrastructure of the diaphragmatic sarcomeres was observed under a transmission electron microscope (TEM) (JEM-1230; JEOL Company, Tokyo, Japan), and photomicrographs were taken.

Z-line to Z-line distances (sarcomere length) were measured on TEM images. The samples were then imaged using a TEM at magnifications of 10,000× to 40,000×. The Z-line distances were quantified using image pro plus, which allowed for precise measurement of sarcomere length. A minimum of 100 sarcomeres per experimental group were analyzed to ensure statistical significance, and the average sarcomere length was calculated for each experimental condition.

### 2.6. Immunohistochemistry Staining

Diaphragm tissue samples were processed to the appropriate size, fixed in 4% paraformaldehyde, followed by processing for paraffin embedding. Then, 8 µm thick sections were prepared and processed by deparaffinization, rehydration, and antigen retrieval. After blocking in a commercial goat serum (Meilunbio, Dalian, China), these sections were incubated overnight at 4 °C with ITGB1 primary antibody (1:1000; Proteintech, Wuhan, China). The next day, we used SP Rabbit HRP Kit (CWBIO, Beijing, China) to amplify the signal and then counter-stained with hematoxylin, dehydrated with a graded ethanol series, and cleared with xylene. Images were captured using a confocal microscope (LSM 880, Zeiss, Jena, Germany) and IHC staining signal was quantified by Image pro plus.

### 2.7. Confocal Microscopic Imaging of F-Actin Staining

Following stress stimulation, C2C12 cells were removed from the apparatus and fixed with 4% paraformaldehyde at room temperature for 30 min, then permeabilized with 0.1% Triton X-100 in PBS for 10 min. Cells were incubated in darkness at room temperature for 1–2 h with TRITC Phalloidin (Solarbio, CA1610, Beijing, China) to stain actin filaments. After incubation, cells were washed twice and mounted using antifade medium containing DAPI (Beyotime, P0131, Shanghai, China). Fluorescence images were acquired using a Zeiss LSM 880 confocal microscope.

### 2.8. Sirius Red Staining

Diaphragmatic tissue was fixed in 4% paraformaldehyde fix solution. Then, the tissue was embedded in paraffin and sectioned to 5 μm thickness. After the paraffin sections were dewaxed, morphological changes in the diaphragmatic tissue were detected. Sirius red was used to detect muscle fibrosis. The photomicrographs were captured using a microscope (Leica, DM2500, Wetzlar, Germany).

### 2.9. RNA-Seq Analysis

RNA sequencing (RNA-seq) was conducted on both rabbit diaphragm tissues and mechanically stimulated C2C12 cells. Sequencing of rabbit diaphragm RNA was performed using 2 × 150 bp paired-end sequencing (PE150) on the Illumina NovaSeq™ 6000 platform (LC Bio Technology Co., Ltd., Hangzhou, China). Similarly, C2C12 cell samples subjected to mechanical stimulation were sequenced on the Illumina HiSeq (Tsingke Biotech Co., Ltd., Beijing, China). All sequencing procedures followed standard manufacturer protocols.

For rabbit diaphragm tissue, differentially expressed mRNAs were identified using DESeq2, with thresholds set at fold change >2 or fold change <0.5 and *p* < 0.05. The resulting set of differentially expressed mRNAs was subsequently subjected to Gene Ontology (GO) enrichment analyses.

RNA-seq analysis of mechanically stimulated C2C12 cells revealed 236 novel genes, 207 of which were functionally annotated. Differential expression analysis was performed using DESeq2, with significant DEGs defined as those showing log2 fold change ≥1 and an adjusted *p* ≤ 0.05. The analysis included normalization, dispersion estimation, and multiple-testing correction. GO enrichment analysis identified significantly enriched terms (*p* ≤ 0.05) among DEGs relative to the genomic background.

### 2.10. Quantitative RT-qPCR

Real-time reverse transcriptase polymerase chain reaction (RT-qPCR) was performed to analyze GAPDH mRNA expression, and all data were normalized to GAPDH levels. For miRNA analysis, total RNA was extracted using the mRNA assay protocol, and cDNA was synthesized from 1000 ng of the total RNA using the One Step PrimeScript miRNA cDNA Synthesis Kit (TaKaRa, Dalian, China). The cDNA was diluted with ddH2O and used for reverse transcriptase polymerase chain reaction, as described in TB Green Premix Ex Taq (TaKaRa). For each sample, the 2^−∆∆Ct^ method was used to evaluate the mRNA abundance for target genes, and GAPDH was adopted as the housekeeping gene. The respective primers are shown in Table 1 and Table 2.

### 2.11. Western Blot Analysis

Protein lysates extracted from diaphragm tissues were separated by SDS-PAGE and subsequently transferred to a polyvinylidene fluoride membrane (IPVH00010, Millipore, MA, USA). After blocking with QuickBlock Blocking Buffer (P0252, Beytime, Shanghai, China) for 20 min, membranes were immunoreacted with primary antibody overnight. The primary antibodies were as follows: GAPDH (sc-53,870, 1:1000, Santa Cruz, CA, USA), COL3 (sc-100, 1:1000, Santa Cruz, CA, USA), COL1A1 (ET7109-25, 1:1000, Hua An Biotechnology, Hangzhou, China), TGFβ-1 (A3101, 1:1000, ABclonal, Wuhan, China), ITGB1 (12594-1-AP, 1:2000, Proteintech, Wuhan, China), and α-SMA (ET1607-53, 1:5000, Hua An Biotechnology, Hangzhou, China). After incubation with secondary antibodies (1:5000, Proteintech, Wuhan, China), bands were visible on the membrane with the aid of enhanced chemiluminescence (ECL) reagent. GAPDH was used as a loading control. ImageLab software (Version 6.1) was utilized for quantitative analysis.

### 2.12. Statistical Analysis

Statistical significance between the two groups was calculated with Student’s *t*-test. Comparisons between of three groups were conducted by one-way analysis of variance (ANOVA) and nonparametric tests, and the values are presented as the means ± SDs. A *p* value of less than 0.05 by SPSS 19.0 statistical software was considered statistically significant.

## 3. Results

### 3.1. High PEEP During Mechanical Ventilation Leads to Changes in Respiratory Mechanics and Diaphragmatic Dysfunction in Rabbits

**Figure 1:**
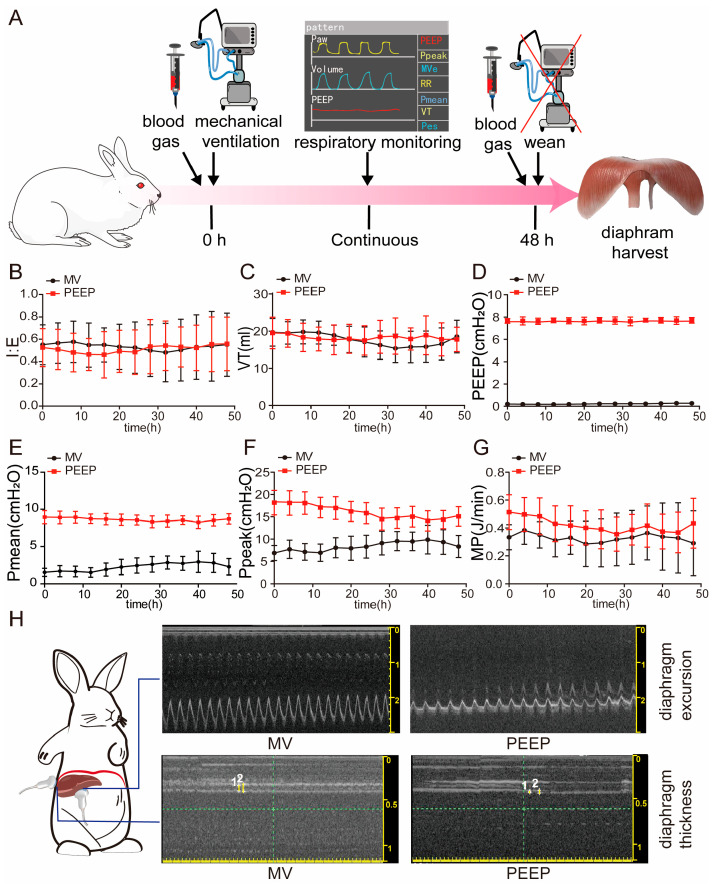
Respiratory parameter monitoring during model establishment. (**A**) Schematic diagram of the mechanical ventilation rabbit model. (**B**) Respiratory parameter monitoring of I:E. (**C**) Respiratory parameter monitoring of VT (mL). (**D**) Respiratory parameter monitoring of PEEP (cmH_2_O). (**E**) Respiratory parameter monitoring of Pmean (cmH_2_O). (**F**) Respiratory parameter monitoring of Ppeak (cmH_2_O). (**G**) Respiratory parameter monitoring of MP without PEEP(J/min). (**H**) Diaphragm ultrasound assessed diaphragm function and movement in mechanically ventilated rabbits, and representative view at the diaphragm of excursion and thickness. Values are represented as the mean ± SD.

The initial body weights of the CON group, the MV group, and the PEEP group showed no statistically significant differences. We carried out a uniform respiratory strategy, with the exception of PEEP, and continuously monitored the process (Figure 1A) of model establishment to ensure sufficient ventilation support and the maintenance of an optimal breathing pattern in the rabbits. During the model establishment, the inspiration/expiration ratio (I:E) in the MV group and the PEEP group was 0.5346 ± 0.02744 and 0.5124 ± 0.03306 (*p* > 0.05), respectively. The tidal volume was 17.76 ± 1.687 mL and 18.30 ± 0.6613 (*p* > 0.05), respectively. PEEP, mean airway pressure (Pmean), and peak inspiratory pressure (Ppeak) in the MV group were significantly lower than in the PEEP group (PEEP: 0.2205 ± 0.03304 cmH_2_O and 7.684 ± 0.03475 cmH_2_O. Pmean: 2.249 ± 0.5288 cmH_2_O and 8.632 ± 0.2240 cmH_2_O. Ppeak: 8.365 ± 1.037 cmH_2_O and 16.02 ± 1.513 cmH_2_O, respectively). Moreover, mechanical power (MP) without PEEP was lower in the MV group than in the PEEP group (MP: 0.3277 ± 0.02868 J/min and 0.4207 ± 0.05026 J/min, respectively) (Figure 1B–G).

As shown in Table 3, the blood gas parameters at the end of the experiment were not significantly different among groups.

Ultrasound results indicated no statistically significant difference in diaphragm excursion and inspiratory/expiratory diaphragm thickness between the PEEP group and the MV group before the application of mechanical ventilation. However, after accepting mechanical ventilation, the PEEP group exhibited a significant decrease in diaphragm excursion and inspiratory/expiratory diaphragm thickness compared to the MV group (*p* < 0.01, *p* < 0.01, *p* < 0.05, respectively) (Figure 1H and Supplementary Figure S1).

### 3.2. Diaphragmatic Fibrosis Was Found in Rabbits After Mechanical Ventilation with PEEP for 48 h

**Figure 2:**
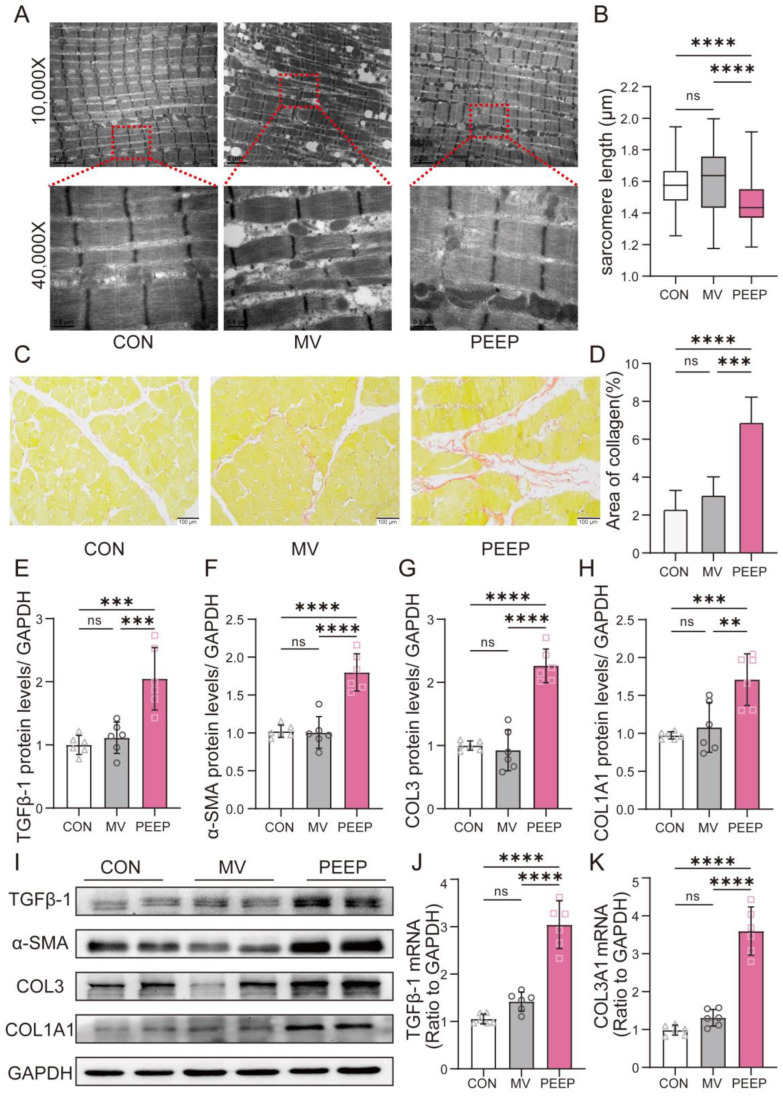
PEEP application during mechanical ventilation contributes to extracellular matrix alterations and collagen deposition and fibrosis associated with TGFβ-1 upregulation. (**A**) Sarcomere length across the three groups. (**B**) TEM revealed alterations in the ultrastructure of the diaphragms. (**C**) Sirius red staining revealed increased collagen fibers in the diaphragms of the PEEP group. All collagen fibers are stained red. The intensity and area of red staining indicate the total amount and density of collagen deposition. Scale bar = 100 μm. (**D**) According to Sirius red staining, area of collagen deposition. (**E**,**F**) The mRNA quantification of collagen 3A1 and TGFβ-1. (**G**–**K**), Diaphragm protein lysates of the three groups were detected by Western blotting, and the expression of different fibrosis0related indicators (TGFβ-1, α-SMA, COL3, and COL1A1) was quantified.The original Western blot images are provided in Supplementary Figure S2. Values are represented as the mean ± SD (** *p* < 0.01, *** *p* < 0.001, **** *p* < 0.0001).

As shown in Figure 2B, TEM observations revealed that diaphragms in the CON group rabbits exhibited a normal myofibrillar ultrastructure with no anomalies in mitochondrial morphology. We observed that the sarcomere length in the PEEP group was significantly shorter compared to the CON and MV groups (1.467 ± 0.1406 μm vs. 1.581 ± 0.1330 μm vs. 1.598 ± 0.1984 μm, respectively, *p* < 0.0001) (Figure 1A). The ultrastructure of diaphragms in the MV and PEEP group was altered, displaying myofibril fragmentation with a large interfibrillar space and sarcomere disruption at a higher magnification (40,000×). Regarding sarcomere length across the three groups, the PEEP group was shorter than other groups.

We confirmed that PEEP application during mechanical ventilation induced extracellular matrix remodeling in the diaphragm. The collagen fiber staining in the PEEP group was stronger than that in the MV group (3.062 ± 1.027 and 6.906 ± 1.388, respectively, *p* < 0.0001) (Figure 2A,B). By further probing our data, we found some differentially regulated genes associated with collagen, as shown in Figure 2C,D. The COL3A1 and TGFβ-1 mRNA expression levels were further verified to be increased in the PEEP group compared to the MV group (COL3A1: 1.301 ± 0.3445 and 2.809 ± 1.298, respectively, *p* = 0.001; TGFβ-1: 1.488 ± 0.2671 and 2.424 ± 0.8925, respectively, *p* < 0.05). Similar results were found by Western blotting: the protein expression levels of TGFβ-1, α-SMA, COL 3, and COL1A1 were significantly increased in the PEEP group compared to the MV group (TGFβ-1: 0.9498 ± 0.1266 and 1.598 ± 0.1623, respectively, *p* < 0.01; α-SMA: 0.9635 ± 0.087 and 1.544 ± 0.1071, respectively, *p* < 0.0001; COL 3: 0.8691 ± 0.1304 and 2.015 ± 0.3391, respectively, *p* < 0.01; COL1A1: 1.130 ± 0.4736 and 2.725 ± 1.993) (Figure 2E–I). These findings collectively indicate that the application of PEEP during mechanical ventilation may lead to extracellular matrix alterations and collagen deposition and fibrosis associated with TGFβ-1 upregulation.

### 3.3. Diaphragm Fibrosis in Mechanical Ventilation with PEEP Application Is Associated with Mechanical Force Stimulation and Activated ITGB1

**Figure 3:**
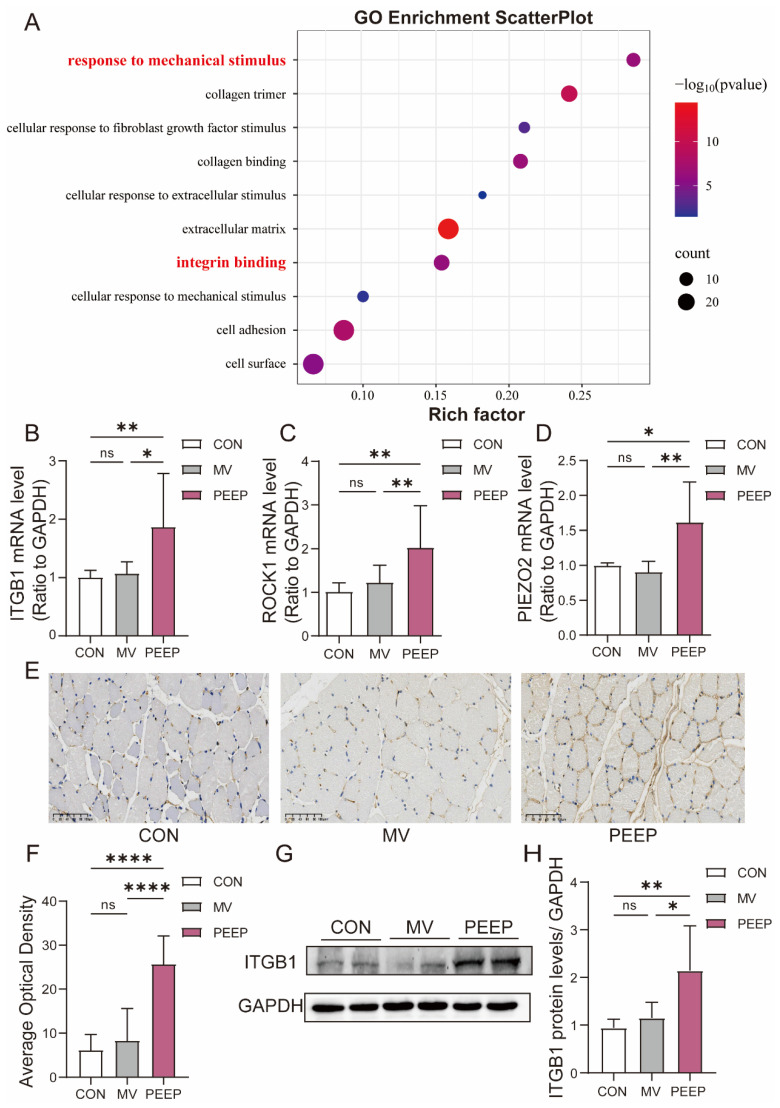
Diaphragm fibrosis in mechanical ventilation with PEEP application is associated with mechanical force stimulation, especially integrin proteins—ITGB1. (**A**) Bubble chart of the significant categories for GO enrichment of the MV group and the PEEP group (*n* = 3). Red font designation: indicates GO terms with significant enrichment in mechanical stimulus response and integrin binding pathways. (**B**–**D**) The diaphragm of the two groups was subjected to qPCR to analyze ITGB1/ROCK1 and PIEZO2. (**E**,**F**) Diaphragmatic tissue sections were subjected to immunohistochemistry staining to analyze the expression of ITGB1. Scale bar = 100 μm. (**G**,**H**) Diaphragm protein lysates of the three groups were detected by Western blotting, and the expression of ITGB1 was quantified. The original Western blot images are provided in Supplementary Figure S3. Values are represented as the mean ± SD (ns: Not significant, * *p* < 0.05, ** *p* < 0.01, **** *p* < 0.0001).

Based on previous RNA-seq results from animal studies, Gene Ontology (GO) analysis of differentially expressed genes indicated that significantly enriched GO terms were associated not only with the extracellular matrix and fibrosis, but also with responses to mechanical stimulus and integrin binding (Figure 3A). Therefore, diaphragm fibrosis in mechanical ventilation with PEEP application may result from changes in the biomechanical environment of the diaphragm, which activate channels related to mechanical mechanics.

We conducted RT-qPCR analysis on the diaphragm tissue of New Zealand rabbits subjected to mechanical ventilation with PEEP. The data revealed that mechanically sensitive molecules such as ITGB1, ROCK1, and PIEZO2 (ITGB1: 1.076 ± 0.2188 and 1.874 ± 0.9339, respectively, *p* < 0.05; ROCK1: 1.315 ± 0.4098 and 1.901 ± 0.8700, respectively, *p* < 0.01; PIEZO2: 0.9079 ± 0.1711 and 1.617 ± 0.5940, respectively, *p* < 0.01) (Figure 3B–D) were significantly upregulated at the transcriptional expression levels.

In conjunction with the animal RNA-seq GO analysis, we performed immunohistochemistry (IHC) staining for ITGB1. By assessing the staining intensity, we calculated the average optical density within different groups. The results indicated that the PEEP group exhibited the most intense ITGB1 staining (8.347 ± 7.551 and 25.73 ± 6.644, respectively, *p* < 0.0001) (Figure 3E,F). Additionally, Western blot analysis demonstrated that ITGB1 expression was significantly higher in the PEEP group compared to MV groups (1.076 ± 0.2188 and 1.874 ± 0.9339, respectively, *p* < 0.0001) (Figure 3G,H).

### 3.4. ITGB1 Expression and Cellular Morphological Changes Under Mechanical Stimulation

**Figure 4:**
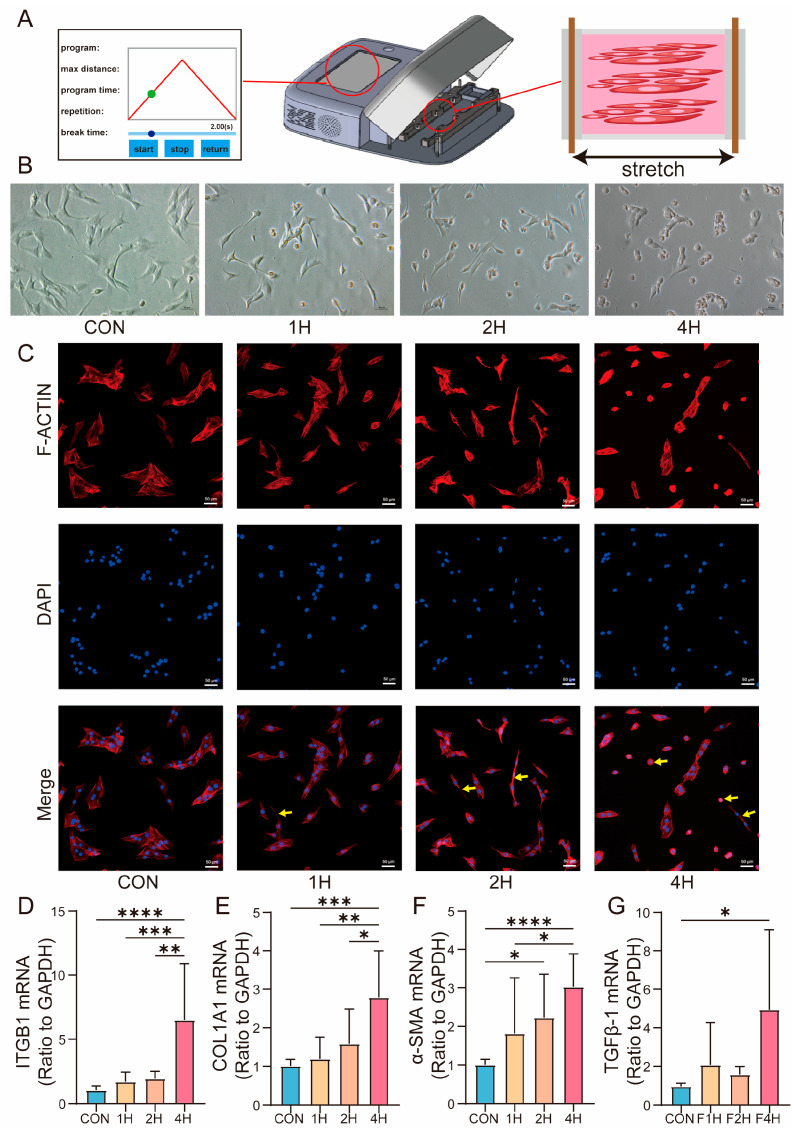
ITGB1, fibrosis-related markers’ expression, and cellular morphological changes in response to mechanical stimulation. (**A**) Schematic representation of the cell stretching model. (**B**) Bright-field images of C2C12 subjected to mechanical stretching for varying durations. Scale bar = 10 μm. (**C**) F-actin staining of C2C12 using phalloidin after mechanical stretching for different durations. The yellow arrow indicates changes in the cytoskeleton. Scale bar = 50 μm. (**D**–**G**) mRNA expression of ITGB1, COL1A1, α-SMA, and TGFβ-1 of C2C12 experiencing 15% elongation of cyclic tensile stretch for 0, 1, 2, and 4 h. Values are represented as the mean ± SD (* *p* < 0.05, ** *p* < 0.01, *** *p* < 0.001, **** *p* < 0.0001).

The diaphragm of rabbits resides in mechanically dynamic environments, particularly during MV with PEEP. To investigate the effects of mechanical forces on cellular behavior, we applied mechanical stretching to C2C12 myoblasts at varying durations (Figure 4A).

In the 1 h stretching condition (1H), cells displayed an initial elongation along the direction of force application, with early signs of cytoskeletal reorganization. Specifically, actin filaments were repositioned, and the cells assumed an elongated morphology, reflecting an adaptive response to the applied mechanical tension. As the stretching duration was extended to 2 h (2H), more pronounced morphological changes were observed. The cells exhibited a more defined “string-like” shape, and a notable increase in cord-like cellular aggregation occurred, suggesting that longer stretching led to a stronger mechanical response. After 4 h of continuous stretching (4H), the cells demonstrated a reduction in elongation. The cytoskeleton showed further deformation, and cells exhibited signs of shrinkage and contraction, indicating a brief morphological collapse. Despite these changes, no cell death was observed, and the cellular morphology was reversible upon release of mechanical tension (Figure 4B,C).

Furthermore, we detected the expression levels of ITGB1 and fibrosis-related mRNAs at different timepoints during mechanical stretching. Notably, after 4 h of stretching, ITGB1 and the expression of fibrotic markers, such as COL1A1, α-SMA, and TGFβ-1, were significantly increased, correlating with an early stage of cellular mechanical response (Figure 4D–G). (CON vs. 1H vs. 2H vs. 4H) (ITGB1: 1.044 ± 0.3321 vs. 1.706 ± 0.7370 vs. 1.938 ± 0.5739 vs. 6.483± 1.128; COL1A1: 1.012 ± 0.1663 vs. 1.188 ± 0.5690 vs. 1.587 ± 0.8968 vs. 2.226 ± 1.128; α-SMA: 1.008 ± 0.1341 vs. 1.815 ± 1.445 vs. 2.226 ± 1.128 vs. 2.226 ± 1.128; TGFβ-1: 0.9652 ± 0.1658 vs. 2.075 ± 2.188 vs. 1.580 ± 0.4192 vs. 4.926 ± 4.177).

### 3.5. ITGB1-Dependent Modulation of TGFβ-1 Signaling Alters Fibrosis Expression Under Long-Term Mechanical Stress

**Figure 5:**
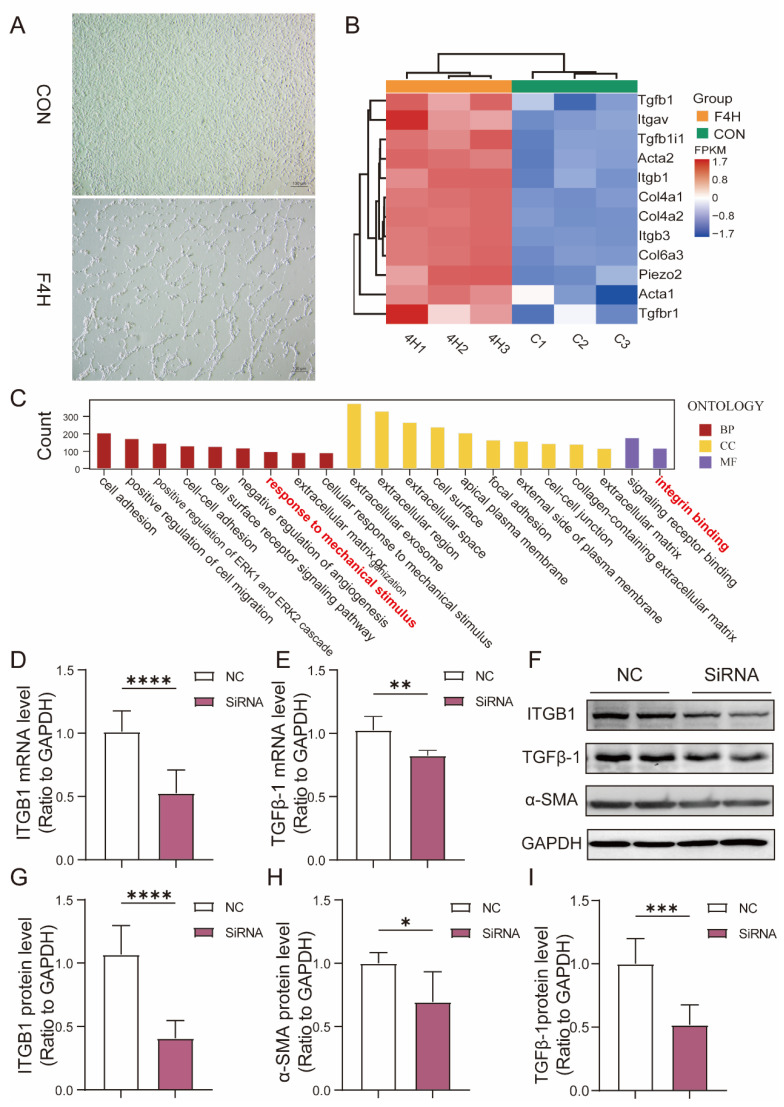
ITGB1-dependent modulation of TGFβ-1 signaling regulates fibrosis-related gene expression under prolonged mechanical stress. (**A**) Bright-field images of C2C12 subjected to mechanical stretching for CON vs. 4H, Scale bar = 100 μm. (**B**) Heatmap showing differential gene expression related to integrins and fibrosis in the CON and 4H groups (*n* = 3). (**C**) GO enrichment analysis of genes associated with mechanical stimuli, integrin binding, extracellular matrix, and fibrosis in the CON and 4H groups, presented as a column chart (*n* = 3). (**D**,**E**) mRNA expression levels of ITGB1 and TGFβ-1 following ITGB1 knockdown by siRNA. (**F**–**I**) Protein expression of ITGB1, α-SMA, and TGFβ- 1 after ITGB1 knockdown by siRNA. The original Western blot images are provided in Supplementary Figure S4. Values are represented as the mean ± SD (* *p* < 0.05, ** *p* < 0.01, *** *p* < 0.001, **** *p* < 0.0001).

Based on previous results from a cellular mechanical stretching model, under low magnification, after 4 h of stretching, clear strand-like adhesion between cells was observed, and the cells themselves exhibited noticeable shrinkage (Figure 5A). RNA-seq analysis comparing the CON group and the 4H group revealed that ITGB1, TGFβ-1, and several fibrosis-related genes were upregulated in the 4H group, consistent with our findings from the rabbit MV model (Figure 5B). Interestingly, GO analysis of differentially expressed genes showed significant enrichment of pathways associated with responses to mechanical stimulus, integrin binding, the extracellular matrix, and fibrosis, which aligned with the results from the animal experiments (Figure 5C).

ITGB1 is known to translocate to the nucleus upon mechanical stimulation, where it activates downstream signaling pathways, including TGF-β1 signaling. To investigate whether ITGB1 modulates the expression of fibrosis-related genes, cells were treated with an ITGB1 inhibitor. As shown in Figure 5D and Figure 5G, ITGB1 mRNA (CON vs. SiRNA: 1.012 ± 0.1642 vs. 0.5254 ± 0.1845, respectively, *p* < 0.0001) and protein expression (CON vs. SiRNA: 1.068 ± 0.2293 vs. 0.4073 ± 0.1388, respectively, *p* < 0.0001) was successfully knocked down by using siRNA. Inhibition of ITGB1 led to a significant reduction in the TGFβ-1 mRNA expression levels (Figure 5E) (CON vs. SiRNA: 1.025 ± 0.1080 vs. 0.8244 ± 0.04123, respectively, *p* < 0.01) and proteins of TGFβ-1 and α-SMA (Figure 5F–I) (α-SMA: 1.000 ± 0.08476 vs. 0.6943 ± 0.2394; TGFβ-1: 1.000 ± 0.1994 vs. 0.5185 ± 0.1573), indicating that ITGB1 plays a critical role in mediating mechanical force-induced fibrotic responses.

## 4. Discussion

The diaphragm is the most important respiratory pump, driving alveolar ventilation. It is a complex and heterogeneous tissue primarily composed of multinucleated muscle fibers that facilitate movement [13]. The thickness of the diaphragm depends on the proportion of muscle fibers and extracellular matrix (ECM) components, such as collagen, present in the tissue [5,14]. Moreover, the diaphragm ECM exhibits a certain degree of plasticity. Studies on diaphragms from COVID-19 patients who underwent mechanical ventilation have shown that while diaphragmatic fiber atrophy occurs [3,15], there is also an accumulation of ECM. The spaces left behind by atrophied muscle fibers are filled by ECM, and imaging studies suggest that the observed diaphragm thickness remains unchanged or even increases, which may be attributed to diaphragmatic replacement fibrosis. In the present study, diaphragmatic tissue samples from rabbits subjected to 48 h of high-PEEP mechanical ventilation (Figure 1A–G) similarly exhibited abnormal ECM accumulation [16], with the high-PEEP group showing more pronounced ECM deposition than the mechanical ventilation-only group (Figure 2C–K).

When muscle cells are subjected to external mechanical forces and changes in the ECM, integrins act as the bridge between the ECM and intracellular signaling pathways [17]. They play a crucial role in transmitting and modulating the signals from external mechanical forces [18]. Among these, ITGB1, a β1 subunit integrin, has been identified as a key player in this process. Several studies have demonstrated that ITGB1 in muscle cells can activate TGFβ- 1 [19,20,21]. In further research conducted by our team, we found that integrin expression was significantly upregulated in the high-PEEP mechanical ventilation rabbit model (Figure 3A–H).

Under mechanical stimulation, ITGB1 undergoes conformational changes and clustering, transmitting signals through focal adhesion kinase (FAK) and Rho GTPase pathways (such as RhoA/ROCK), thereby directly modulating actin filament assembly, stress fiber formation, and cellular contractility. Studies have demonstrated that mechanical stretch induces rearrangement of the actin cytoskeleton via the ITGB1–RhoA–ROCK axis, consequently influencing cell morphology, migration, and differentiation [22,23,24]. Our study consistently observed that mechanical stimulation induces significant cytoskeletal alterations in cells, with the degree of deformation progressively intensifying as the duration of stretch exposure increases. Notably, these structural changes were accompanied by a corresponding upregulation of ITGB1 expression, suggesting a potential role of ITGB1 in mediating the cellular mechanoresponse (Figure 4A–D).

TGFβ-1 plays a crucial role in the initiation and progression of fibrosis, serving as a key mediator in the fibrotic process. It is stored in the ECM as an inactive precursor protein, which is bound by the Latency-Associated Peptide (LAP) to prevent its binding to receptors, rendering TGFβ- 1 biologically inactive. The activation of TGFβ-1 requires the release of the active form, which occurs when integrins bind to the RGD sequence within the precursor protein structure. Mechanical force is a potent activator of this pathway [19].

Under TGFβ-1 stimulation, cells express a range of growth factors and cytokines, such as α-SMA. These factors bind directly to their receptors, triggering the activation of downstream effector proteins, such as Smad3. This signaling cascade amplifies the fibrotic response, driving the pathological changes associated with fibrosis [25].

The findings of this study provide important clinical insights into the management of mechanical ventilation, particularly in the context of high-PEEP settings, which are commonly used to improve oxygenation in critically ill patients. While high PEEP has been shown to enhance lung compliance and prevent alveolar collapse, previous studies have also shown that prolonged mechanical ventilation with high PEEP can induce lung injury through mechanisms such as volutrauma, barotrauma, and biotrauma, potentially promoting or exacerbating pulmonary fibrosis [26,27,28,29]. Existing research has primarily focused on lung-protective ventilation strategies and ventilator-induced lung injury (VILI); however, these results underscore the importance of carefully balancing PEEP settings in clinical practice to achieve dual protection for both the lungs and the diaphragm. Our research highlights the potential risk of diaphragmatic injury and subsequent fibrosis induced by prolonged mechanical ventilation at high PEEP levels. The activation of ITGB1 and TGFβ-1 signaling pathways, as demonstrated in this study, plays a central role in driving diaphragmatic fibrosis under these conditions [30,31].

Given the critical role of the diaphragm in respiration, strategies aimed at minimizing diaphragmatic damage are essential for improving patient outcomes, particularly in patients requiring long-term mechanical ventilation. Clinically, a more nuanced approach to ventilation settings may be needed to balance the benefits of high PEEP with potential harm to the diaphragm. For instance, ventilator strategies that incorporate periodic reductions in PEEP or the use of alternative strategies such as diaphragm-protective ventilation could help reduce mechanical stress on the diaphragm, thereby minimizing the risk of fibrosis and preserving diaphragmatic function.

Additionally, interventions that target the molecular pathways identified in this study, specifically the ITGB1 and TGFβ-1 axis, could offer promising therapeutic avenues. For example, pharmacological inhibitors of integrin signaling or TGFβ-1 activation may help prevent or mitigate diaphragmatic fibrosis in patients receiving high-PEEP ventilation. The development of such therapies could provide a means of protecting the diaphragm from ventilator-induced injury and improving overall respiratory function in critically ill patients.

Our study has shown that both ITGB1 and TGFβ-1 play central roles in mediating the fibrotic response in the diaphragm under mechanical stress [20], with ITGB1 acting as a mechanosensor that activates TGFβ-1 signaling pathways [32]. The activation of TGFβ-1, in turn, triggers the deposition of extracellular matrix (ECM) components, promoting fibroblast proliferation and myofibroblast differentiation, which ultimately leads to fibrosis and impaired diaphragmatic function (Figure 5B–I).

Future research should explore the broader implications of ITGB1 and TGFβ-1 in fibrosis beyond the diaphragm, particularly in other organs that are commonly affected by mechanical ventilation and critical illness, such as the lungs and heart. In the context of lung fibrosis, for example, investigating the role of ITGB1 and TGFβ-1 in the development of VILI and acute respiratory distress syndrome (ARDS) could provide valuable insights into how mechanical stress leads to fibrotic changes in lung tissue [32]. Clinical studies have demonstrated that high PEEP can alter respiratory drive and breathing effort, with these effects closely linked to lung recruitability. In highly recruitable lungs, increased PEEP reduces the activity of pulmonary C fibers and irritant receptors, which are typically activated by consolidation or atelectasis, thereby decreasing respiratory drive. However, when PEEP fails to alleviate lung consolidation, it may instead impair diaphragm geometry and contraction strength by increasing end-expiratory lung volume [33]. Our further clinical research can investigate whether PEEP can modulate respiratory mechanics to influence diaphragm mechanical stress and mitigate fibrosis following mechanical ventilation.

Understanding how these molecules contribute to alveolar and interstitial fibrosis could help identify new therapeutic targets to prevent or treat respiratory failure in ventilated patients.

Finally, it is crucial to investigate the timing and duration of therapeutic interventions targeting ITGB1 and TGFβ-1. Given the dynamic nature of fibrosis, understanding the optimal windows for intervention—whether early in the fibrotic process to prevent its onset or later to reverse established fibrosis—could significantly impact clinical outcomes. Advanced preclinical models that replicate human mechanical ventilation conditions and fibrotic disease could be valuable tools in assessing the efficacy of these potential therapies.

Despite the valuable insights provided by this study, several limitations need to be addressed in future research.

First, we did not collect animal CT imaging data under high-PEEP mechanical ventilation conditions, which would have provided a more direct and visual representation of diaphragmatic changes. Although we were unable to perform these imaging analyses in the current study, the existing literature supports our findings on diaphragm alterations under high PEEP. Furthermore, we have initiated clinical data collection on diaphragm changes in patients receiving high PEEP, which will complement our findings and provide additional evidence for the impact of high PEEP on diaphragmatic function.

Second, while our cell-based experiments validated the effects of mechanical stretching at various durations, we did not simulate other mechanical forces, such as compression or multi-directional stress, which may also play significant roles in the fibrotic process. Exploring different mechanical stimuli and their influence on ITGB1 activation and TGFβ-1 signaling in muscle cells will be critical for understanding the full spectrum of mechanical stress-induced fibrosis.

Third, in our investigation of ITGB1, we only performed transient ITGB1 knockdown experiments rather than stable full knockout genetic experiments, either in vitro or in animal models. Furthermore, we performed functional characterization following ITGB1 knockout and overexpression cell lines to systematically assess how ITGB1 modulates mechanotransduction pathways—including cytoskeletal dynamics and SMAD2/3 phosphorylation—in response to mechanical stress. In future work, employing ITGB1-knockout rabbit models subjected to high-PEEP mechanical ventilation would more conclusively demonstrate the specific role of ITGB1 in VIDD. These additional experiments will provide further validation of the role of ITGB1 in diaphragmatic fibrosis and clarify its potential as a therapeutic target.

Fourth, the use of healthy rabbits in this study, while enabling a focused investigation into the isolated effects of high PEEP on the diaphragm, limits direct clinical extrapolation. Clinically, mechanical ventilation is typically applied in patients with pre-existing respiratory pathologies such as ARDS, where concomitant inflammatory and structural alterations may modulate ventilator-induced diaphragmatic injury. To improve translational relevance, future studies should validate these mechanisms in disease models—such as experimentally induced ARDS.

In conclusion, while this study presents significant findings, future experiments will be designed to overcome these limitations and provide a more comprehensive understanding of the mechanisms driving diaphragmatic fibrosis under high-PEEP ventilation.

## 5. Conclusions

We emphasized the critical role of ITGB1 in regulating mechanical stress-induced fibrosis of the diaphragm during mechanical ventilation with high-PEEP application. Our findings demonstrated that ITGB1 acts as a central mechanosensor, mediating the fibrotic response by activating the TGFβ-1 signaling pathway. This activation of TGFβ-1 promotes the accumulation of extracellular matrix proteins, fibroblast proliferation, and tissue remodeling, ultimately contributing to diaphragmatic fibrosis.

The interaction between these molecules amplifies the fibrotic signaling cascade, suggesting that targeting either ITGB1 or TGFβ-1 may offer therapeutic potential for mitigating diaphragm injury and dysfunction in mechanically ventilated patients, particularly those requiring high-PEEP settings.

## Figures and Tables

**Table 1:** Primers used in quantitative RT-qPCR study.

Rabbit Gene	Forward Primer	Reverse Primer
TGF-β1	CTGGAACGGGCTCAACATCT	CAGGTCCTTGCGGAAGTCAA
COL1A1	ACCACTGCAAGAACAGCGTA	TCGTGGAGGACAGTGTAGGT
COL3A1	AACAATGGTAGTCCTGGCGG	CACCGTTCTTACCGGGTTCA
Piezo2	CTCAACGTGGGAAAAGACA	CCAGACTCGCAATGAACA
GAPDH	TAAGAGCCCTCAAACCACCG	AAGAGGGGCAGATTCTCAGC
ITGB1-F	CCAGTGCCGAGCCTTCAATA	CAGCAGTCGTCCACATCCTT
ROCK1-F	CTCGCGGAGTAAGTTGGTTGA	CAAAGCATCCAATCCATCCAGC

**Table 2:** Primers used in quantitative RT-qPCR study.

C2C12 Gene	Forward Primer	Reverse Primer
TGF-β1	CCTGAGTGGCTGTCTTTTGA	CGTGGAGTTTGTTATCTTTGCTG
ACTA2	TGAGCGTGGCTATTCCTTCG	AGCGTTCGTTTCCAATGGTG
COL1A1	ACAGTCGCTTCACCTACAGC	GGGTGGAGGGAGTTTACACG
COL3A1	ACGTAAGCACTGGTGGACAG	CAGGAGGGCCATAGCTGAAC
GAPDH	TGTCAAGCTCATTTCCTGGT	TAGGGCCTCTCTTGCTCAGT
ITGB1	TTCAGACTTCCGCATTGGCT	CAGCCAATCAGCGATCCACA

**Table 3:** Blood gas analysis in three groups.

	CON	MV	PEEP	*p*
pH	7.39 ± 0.02	7.39 ± 0.04	7.37 ± 0.08	0.70
PaCO_2_ (mmHg)	38.99 ± 3.49	36.68 ± 5.50	36.65 ± 7.00	0.99
PaO_2_ (mmHg)	89.49 ± 12.98	85.77 ± 22.38	75.60 ± 15.13	0.42
Lac (mmol/L)	2.40 ± 0.71	4.40 ± 3.26	4.90 ± 2.10	0.78
HCO^3−^ (mmol/L)	22.65 ± 3.48	21.80 ± 1.85	20.92 ± 2.83	0.57
PaO_2_/FiO_2_ (mmHg)	426.15 ± 61.80	408.41 ± 106.57	360.00 ± 72.03	0.42

Data are expressed as mean ± SD. *p* MV vs. PEEP.

## Data Availability

The raw sequencing data generated in this study have been deposited in the Gene Expression Omnibus (GEO) database under the accession number GSE309024.

## Supplementary Materials

The following supporting information can be downloaded at: https://www.mdpi.com/article/10.3390/biom15101466/s1, Figure S1: Diaphragm ultrasound assessed diaphragm function and movement in mechanically ventilated rabbits before and after mechanical ventilation, and before and after weaning; Figure S2: The original WB images of Figure 2I; Figure S3: The original WB images of Figure 3G; Figure S4: The original WB images of Figure 5F.

## Abbreviations

The following abbreviations are used in this manuscript:

ITGB1	Integrin beta-1
MV	Mechanical ventilation
PEEP	Positive end-expiratory pressure
VIDD	Ventilation-induced diaphragm dysfunction
TGFβ-1	Transforming growth factor-β1
ROCK1	Rho-associated, coiled-coil containing protein kinase 1
α-SMA	α-smooth muscle actin
EELV	End-expiratory lung volume
LAP	Latency-Associated Peptide
PETCO_2_	End tidal CO_2_ pressure
SBT	Spontaneous breathing trial
RR	Respiratory rate
VT	Tidal volume
VE	Total minute ventilation
FBS	Fetal bovine serum
TEM	Transmission electron microscope
GO	Gene ontology
KEGG	Kyoto encyclopedia of genes and genomes
RT-qPCR	Real-time reverse transcriptase polymerase chain reaction
ECL	Enhanced chemiluminescence
ANOVA	One-way analysis of variance
Pmean	Mean airway pressure
Ppeak	Peak inspiratory pressure
MP	Mechanical power
ECM	Extracellular matrix
VILI	Ventilator-induced lung injury
ARDS	Acute respiratory distress syndrome
