# Supplementary material for: High PEEP Activates ITGB1, Inducing Diaphragm Fibrosis During Prolonged Mechanical Ventilation
Source: Biomolecules. 2025 Oct 16;15(10):1466. doi: 10.3390/biom15101466 (PMC12562615; doi:10.3390/biom15101466)
Supplement: Supplementary file 1 [file biomolecules-15-01466-s001.zip › biomolecules-3861667-supplementary.pdf]

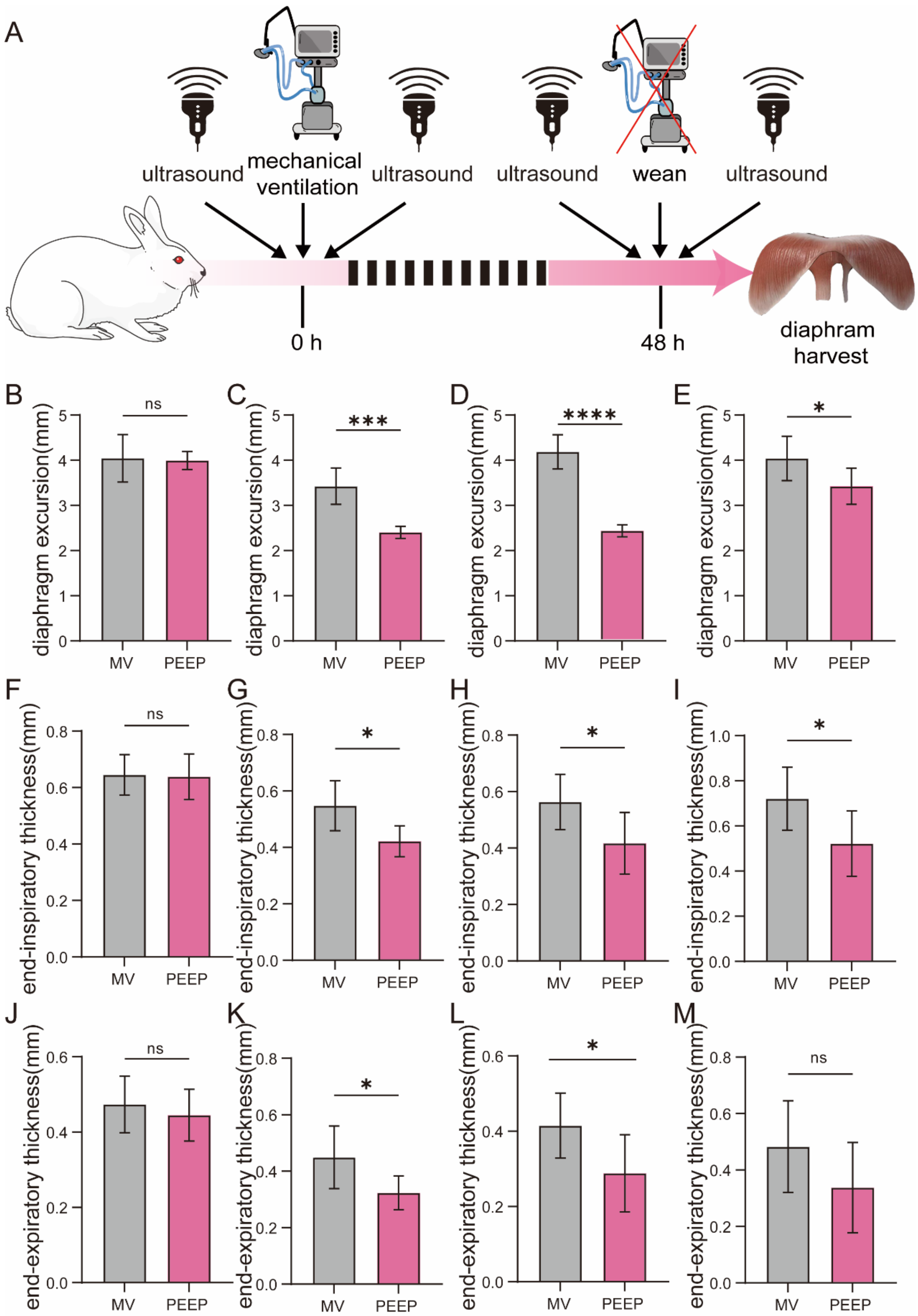

Figure S1. Diaphragm ultrasound assessed diaphragm function and movement in mechanically ventilated rabbits before and after mechanical ventilation, and before and after weaning. A Timeline for the Use of Diaphragm Ultrasound. B-E diaphragm excursion during different mechanical ventilation time. F-I end-inspiratory during different mechanical ventilation time. J-M end-expiratory during different mechanical ventilation time. Values are represented as the mean ± SD (*p < 0.05, **p < 0.01)

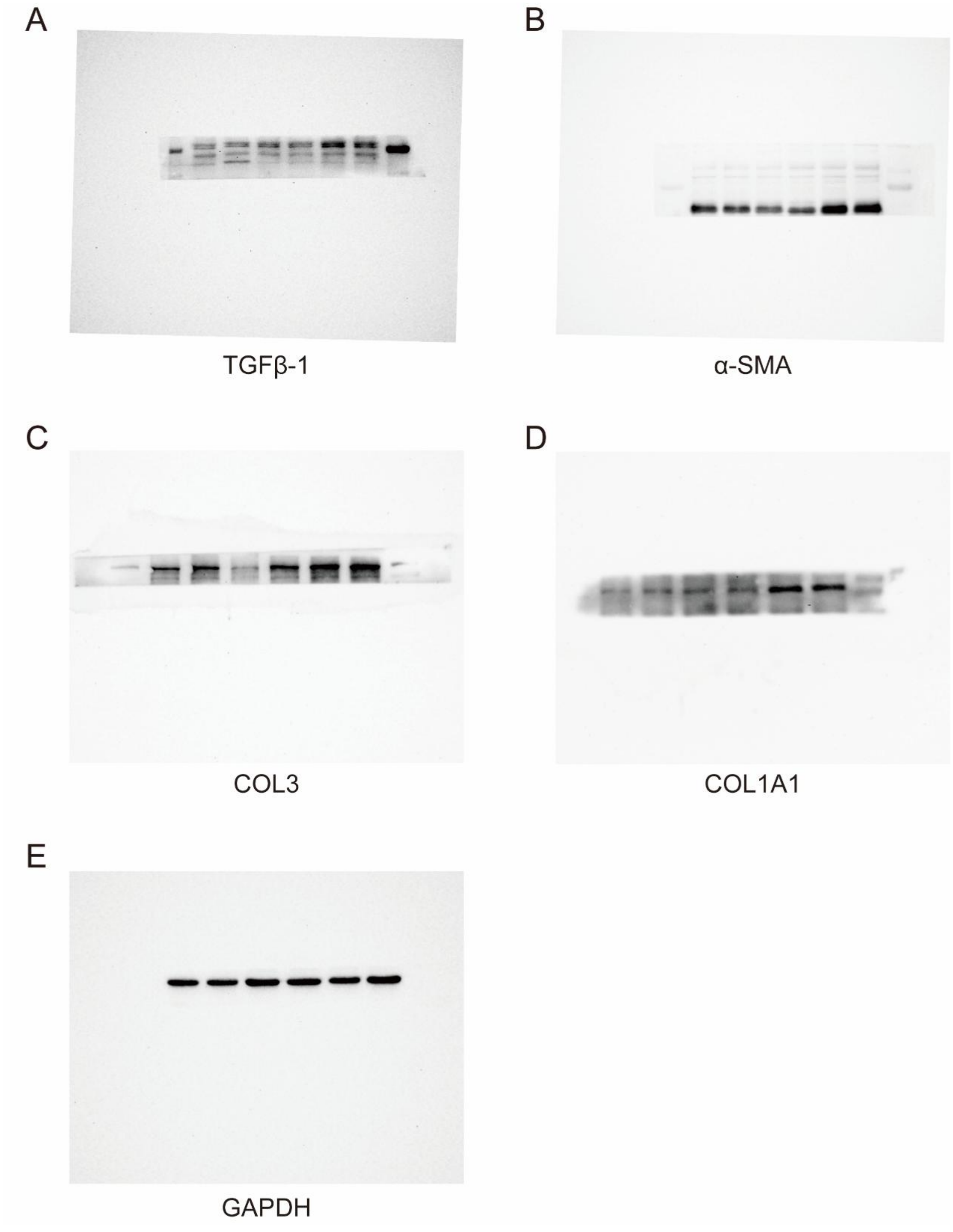

Figure S2. The original WB images of Fig.2 I.

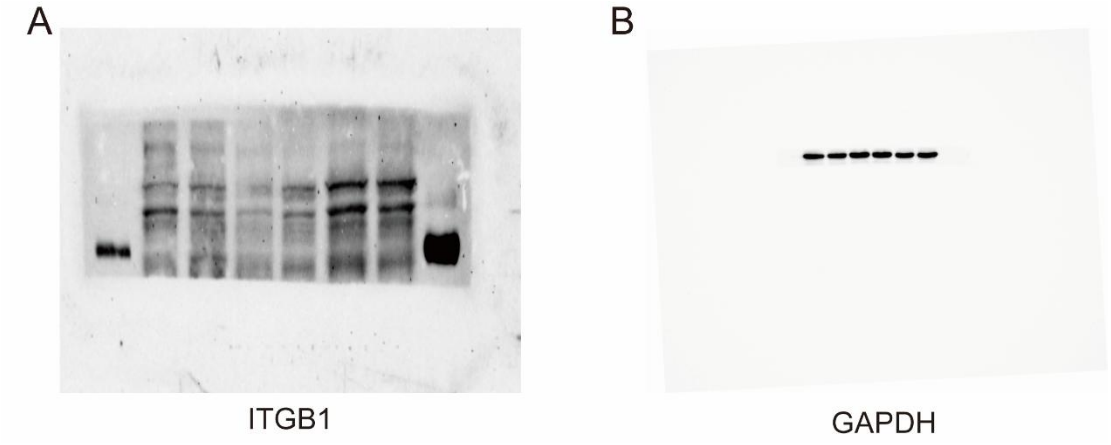

Figure S3. The original WB images of Fig.3 G.

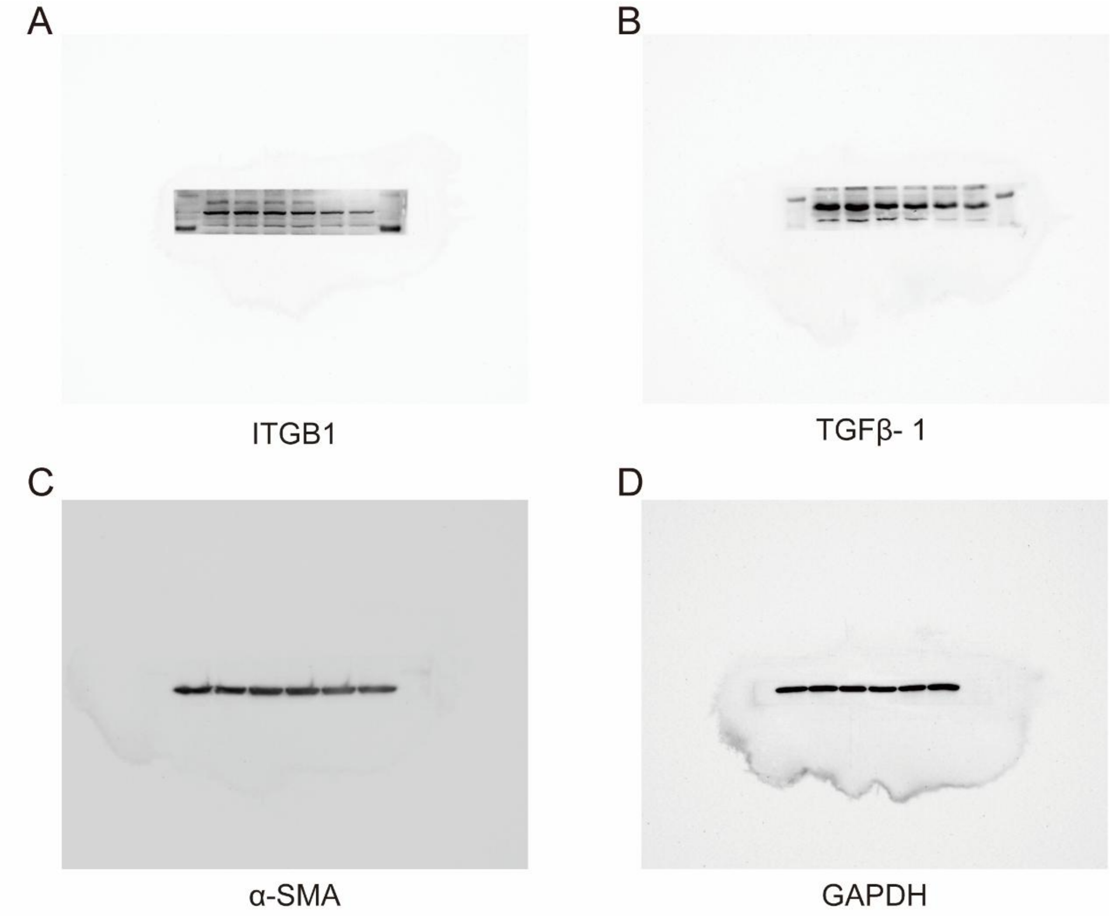

Figure S4. The original WB images of Fig.5 F. The Western blot images presented in this article are representative results from the NC and siRNA groups. The original full-length blots included the CON, NC, and siRNA groups.
